# Investigation of Uncovering Molecular Mechanisms of Alcohol-Induced Female Infertility—A Rational Approach

**DOI:** 10.1007/s43032-024-01692-8

**Published:** 2024-11-01

**Authors:** Natasha Sura Anak Lubau, Neevashini Chengebroyen, Vetriselvan Subramaniyan

**Affiliations:** 1 Pharmacology Unit, Jeffrey Cheah School of Medicine and Health Sciences, Monash University Malaysia, Subang Jaya, Malaysia; 2 Department of Medical Sciences, School of Medical and Life Sciences, Sunway University, Jalan University, Bandar Sunway, 47500 Selangor Darul Ehsan Malaysia

**Keywords:** Alcohol consumption, Mortality, Risks, Mechanism, Female infertility

## Abstract

This study aimed to investigate the molecular mechanisms by which chronic alcohol consumption impacts female infertility, highlighting significant societal implications. By conducting a comprehensive literature review, we examined existing evidence on the association between long-term alcohol use and female reproductive health. Relevant studies were identified through systematic searches of electronic databases and key journals. We synthesized information on the molecular pathways affected by alcohol consumption, with particular emphasis on oxidative stress, inflammation, and hormonal disruptions. Additionally, we reviewed efforts to address alcohol-related health issues, including public health interventions, regulatory measures, and educational initiatives. Our study found strong evidence linking chronic alcohol consumption to increased mortality rates and a range of preventable diseases globally. Alcohol's effects extend beyond physiological consequences to psychological, social, and economic burdens. Chronic alcohol consumption disrupts hormonal balance and reproductive function, contributing to female infertility. Future research should focus on quantifying mortality risks associated with alcohol consumption, understanding gender-specific patterns in alcohol-related health outcomes, and elucidating the molecular mechanisms underlying female infertility. Addressing these gaps will inform strategies to mitigate the burden of alcohol-induced health issues and promote overall well-being. Collaborative efforts among diverse stakeholders are essential for advancing research agendas and translating findings into effective interventions.

## Exploring the Link Between Excessive Alcohol Intake and Increased Mortality

**Fig.1:**
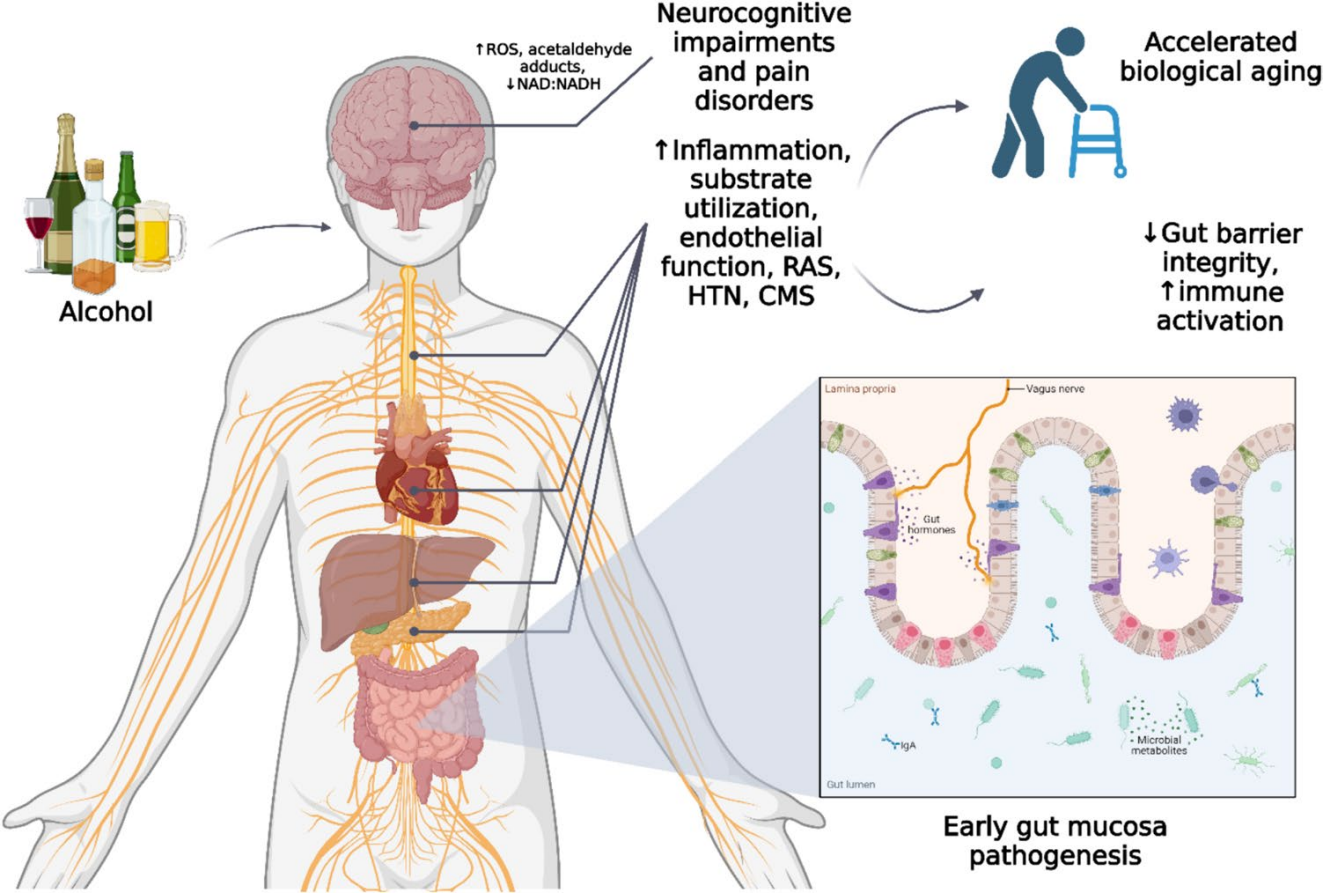
Alcohol interaction. As a result of the deleterious byproducts produced during alcohol metabolism, tissue and cell damage can be accelerated. Several pathophysiological processes, such as epigenetic modifications, growth factor signalling, altered nutritional deficiencies, mitochondrial damage, and oxidative stress, are frequently associated among alcohol metabolisms. These interact in conjunction with inflammation, systemic immune activation, cell senescence, and exhaustion induced by gastrointestinal leakage. As a result, tissue damage is exacerbated and homeostatic mechanisms are disrupted, which raises the risk of developing cardiometabolic syndrome (CMS). CMS is linked to a cluster of target organ dyshomeostasis syndromes, which increases the risk of neurocognitive deficits and pain syndromes, both of which have the potential to accelerate or worsen biological ageing. CMS signifies cardiometabolic syndrome; HTN denotes hypertension; RAS stands for the renin-angiotensin system

Chronic alcohol intake has repeatedly been linked with a higher likelihood of death and a range of health issues [1]. Multiple studies and thorough research have firmly proven a significant correlation between excessive alcohol use and increased death rates. Excessive alcohol consumption can lead to increased mortality in several significant ways [2]. Throughout history, alcoholism has consistently held its place as one of the recognised risk factors for preventable diseases worldwide. Based on research from the World Health Organisation (WHO), 5.3% of global deaths in 2016 were attributed to detrimental alcohol consumption [3]. Alcohol is the primary cause of the progression of numerous chronic diseases, and it significantly amplifies the severity of diseases and diminishes the effectiveness of therapies [4]. Alcohol consumption not only affects the physiological system (Fig. 1) but also has a multitude of adverse consequences on the psychological and social welfare of an individual. Moreover, the inclination towards alcoholism results in substantial economic problems [5]. Furthermore, apart from the diverse array of clinical presentations that ensue from systemic involvement, there exist specific indicators and symptoms that frequently lack specificity and fail to propose or indicate an exact evaluation [6]. Headache, anxiety, vomiting, nausea, delirium, seizures, tremors, elevated body temperature, rapid pulse rate, visual hallucinations, and so forth, are some of these symptoms. These signs and symptoms are not always necessarily associated with illness conditions [1].

### Gender Disparities in Alcohol-Related Mortality: A Closer Look at Risk in Women

**Fig. 2:**
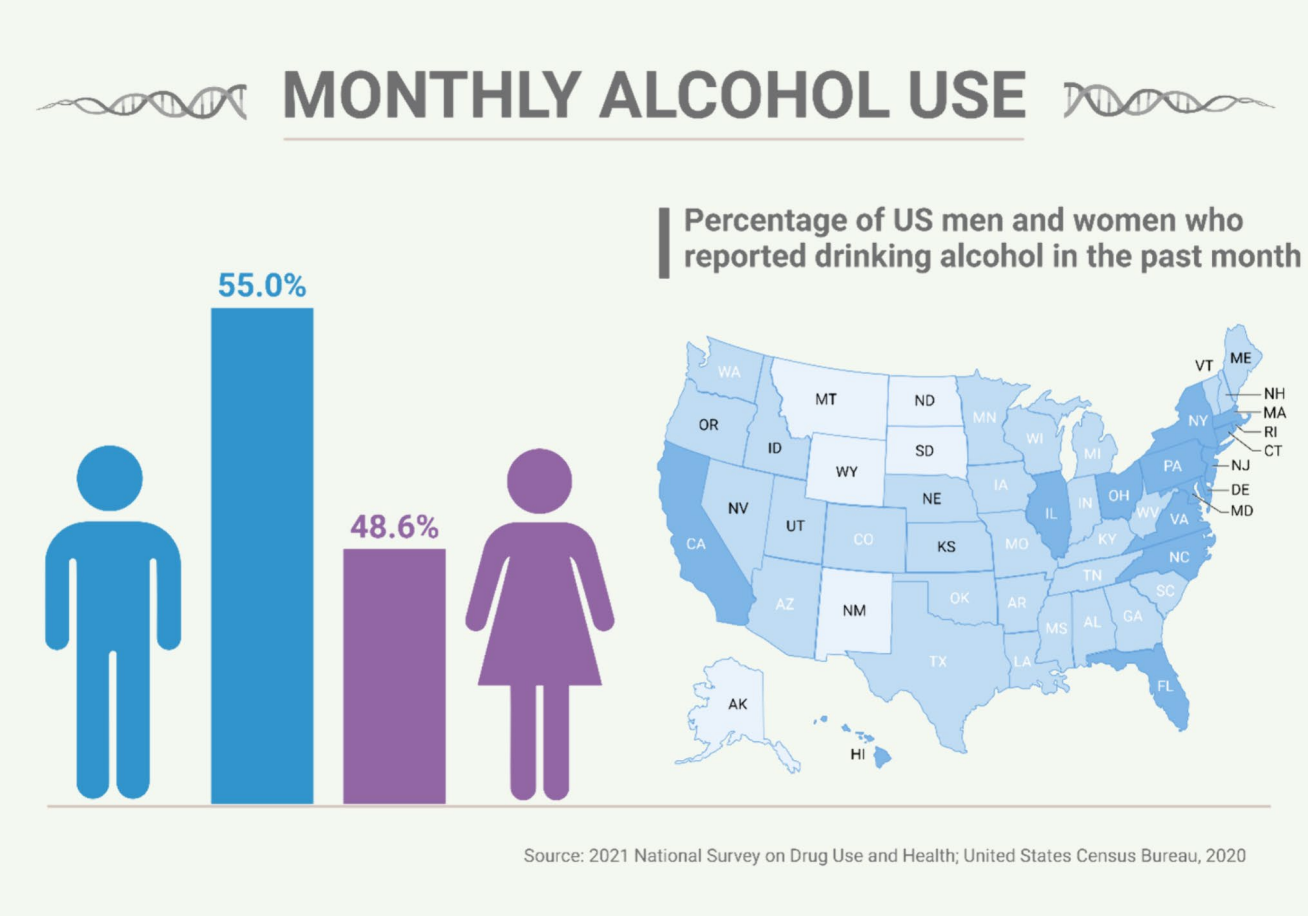
Monthly alcohol use by both men and women in 2020

Research shows that alcohol consumption and abuse among women are on the rise as depicted in (Fig. 2**)** [7, 8]. Although excessive alcohol consumption is harmful to the health of everyone, women are disproportionately impacted by alcohol-related problems [9]. In addition to the 2020–2025 Dietary Guidelines for Americans, women should be aware of these health hazards associated with alcohol usage in order to make informed decisions regarding their use. Adult women who are of legal drinking age might choose to either completely abstain from alcohol or to limit their intake to no more than one drink per day [10]. This amount is not intended to be an average, but rather a daily cap. Lowering alcohol use can lessen its risks, but it won't completely eradicate them. Some people should never drink alcohol at all, such as those who are pregnant or may become pregnant [11]. The study emphasizes the critical need for future research to establish causality through longitudinal and epidemiological studies, highlighting a significant gap in our current understanding of the causal relationship between alcohol consumption and health outcomes. Although existing evidence strongly connects chronic alcohol use to various health issues, such as liver toxicity and female infertility, most studies remain correlational. Longitudinal studies, which track individuals over time, are essential for observing the direct effects of alcohol on health. Similarly, epidemiological studies can reveal causal patterns within populations, clarifying how chronic alcohol consumption contributes to specific health conditions. Additionally, while the study briefly touches on gender disparities in alcohol-related health outcomes, there is a need for deeper exploration into the specific mechanisms linking alcohol consumption to female infertility. This further investigation is crucial for developing targeted interventions and effective public health strategies to mitigate the impact of alcohol-related health problems [12].

### Physiological Differences

In general, women possess a greater percentage of adipose tissue and a lesser percentage of water compared to males [13]. Due to the bodies of women are less able to metabolise alcohol, their blood alcohol concentrations rise with each unit of alcohol they ingest [14]. This may increase the chance of experiencing health problems linked to alcohol use. Several studies show that women experience alcohol-related problems earlier and at lower alcohol intake levels than men do, for a number of reasons [13]. In general, women weigh less than men do. In addition, women have less water per pound than men do, and bodily water serves as the main storage space for alcohol. This suggests that after a male and a woman of equal weight consume an identical amount of alcohol, women are more likely to have elevated blood alcohol concentrations, or blood alcohol content, which increases their risk of harm. For example, studies show that women are more prone than men to develop hangovers and alcohol-induced blackouts at similar alcohol levels [15, 16].

### Metabolism Variation

Alcohol metabolism in women generally differs from that of males as a result of discrepancies in the activity of enzymes involved in alcohol metabolism [17]. This may potentially heighten the vulnerability of an individual to the detrimental consequences of alcohol, such as hepatic impairment. Previous research has documented variations in alcohol metabolism parameters based on gender [18, 19]. In contrast to males, females exhibit reduced activity of alcohol dehydrogenase in the stomach, an enzyme that enhances the bioavailability of ethanol [20]. A 7.3% reduction in the volume of ethanol distribution relative to men causes an increase in blood alcohol concentration among females. In comparison to males, women exhibit a 10% increase in the rate of ethanol oxidation and elimination in the liver, while their gastric emptying rate of alcohol is 42% slower. As a consequence of these disparities, women consume an equivalent amount of alcohol, which elevates their blood alcohol concentration [21–24]. Additionally, disparities between genders have been identified regarding the frequency and volume of alcohol consumption. A cross-cultural investigation reveals that males consume alcohol at a greater frequency and in greater quantities [25, 26]. Conversely, the prevalence of alcohol use disorder, high-risk alcohol consumption, and alcohol use among women has increased substantially over the past decade (16%, 58%, and 84%, respectively), while men have experienced a comparatively lesser surge (7%, 16%, and 35%, respectively) [27, 28]. Gender disparities in a few neurotransmitter systems might significantly influence the likelihood of developing alcohol use disorders [29, 30].

### Pregnancy and Fertility

The consumption of alcohol throughout pregnancy may result in harmful outcomes for the development of the foetus, including the emergence of foetal alcohol syndrome [31]. Alcohol consumption causes distinct considerations and potential repercussions for women who are in the reproduction age group [32]. The optimal level of alcohol consumption for expectant or potentially pregnant women is unknown [33]. Alcohol exposure during pregnancy has the potential to induce physical, cognitive, and behavioural complications in children, all of which may ultimately develop into foetal alcohol spectrum disorders [34]. Additionally, drinking during pregnancy may elevate the likelihood of preterm labour [35].

Jones and Smith (1973) documented a syndrome in foetal subjects exposed to alcohol that manifested as growth retardation, dysfunction of the central nervous system, and facial dysmorphology [36]. It is disconcerting that, notwithstanding reductions in alcohol consumption throughout pregnancy, around 10% of pregnant women persist in consuming alcohol on a monthly basis [37]. Foetal alcohol spectrum disorder (FASD) affects one percent to five percent of American babies, according to recent estimates [38]. Birth weight in babies was found to be lower in a prospective research including almost 31,000 women, even when alcohol consumption was restricted to an average of one drink per day (about 14 grammes of alcohol) [38]. A minimum of 3.5 standard U.S. servings (14 grammes each) of alcohol per week is associated with decreased intelligence quotient (IQ) scores in children as young as eight years old, particularly when those children have one of four genetic variants in the genes accountable for alcohol metabolism [39]. While the detrimental consequences of alcohol exposure appear to be particularly conspicuous during the first trimester, even moderate to low levels of alcohol intake have been associated with cognitive, motor, and developmental setbacks [40, 41]. According to recent studies, alcohol usage by the father before conception may have an impact on the development of the foetus and later alcohol use [42].

### Treatment Disparities

Psychological and sociodemographic differences exist between men and women seeking treatment for AUD [43]. Women face unique challenges in accessing treatment and possess specific needs that differ from those of males undergoing treatment for AUD [44]. Regarding sociodemographic characteristics, AUD-treated women are distinguished from non-participating women. There was a higher prevalence of psychotropic medication use among individuals who held the belief that they did not necessitate treatment [45]. Treatment for AUD in women is impeded by a combination of internal and external barriers [46]. The gender discrepancy in treatment initiation rates can be attributed by diminished recognition of the necessity for treatment, emotions of guilt and shame stemming from the discrepancy between societal expectations and traditional gender norms regarding women with AUD, the presence of depression and other co-occurring disorders, more pronounced inequities in employment, health insurance, and economic prospects in comparison to men, childcare responsibilities, and concerns regarding potential churn [46].

Contemporary gender norms and prevalent misconceptions concerning AUD may contribute to the decreased utilisation of AUD treatment among women, according to recent research. According to research by Lale and colleagues, women were more likely than men to attribute AUD to "bad character" and less likely to attribute AUD to heredity [47]. Furthermore, women articulate apprehensions regarding the possible consequences that may ensue if they disclose an alcohol dependency, such as being viewed as "bad mothers" or losing custody of their children [48]. Likewise, it is observed that women are more likely than males to experience feelings of embarrassment, dread, hopelessness, and the belief that their situation does not warrant AUD treatment [49]. In addition to these interpersonal barriers, women may experience a scarcity of social support in comparison to males when attempting to seek treatment for AUD. There is a higher likelihood that women with AUD are involved in an intimate relationship with another woman who also has the disorder [50]. Additionally, during recovery, women receive less support from their spouses and families [51]. Furthermore, greater logistical barriers are frequently encountered by women when endeavouring to access treatment. The challenges commonly encompass issues related to transportation, restricted availability of nursery, and inadequate insurance coverage [52].

When comparing men and women, it has been observed that women are more likely to seek treatment for AUD in clinical settings specialising in general mental health or substance use. In addition, the likelihood that the court will order them to attend treatment is diminished [53]. Furthermore, women with AUD often cite nonsubstance-related mental health issues and distressing life events as the primary reasons for seeking treatment [54]. The child welfare, legal, and welfare systems provide additional channels through which specific women can obtain treatment for AUD. An initiative to provide primary care physicians, gynaecologists, and psychiatrists with specialised knowledge on how to recognise and refer women with AUD has the potential to reduce the gender gap reflected in the disproportionate number of cases where women seek treatment for AUD. In addition, empirical evidence indicates that caregiving-oriented AUD treatment facilities are generally preferred by women. As a result, it is anticipated that treatment facilities that are easily accessible, suitable for children, and broadly available will also increase the utilisation of services among women who have been diagnosed with AUD [55].

## Complex Molecular Mechanisms Behind Alcohol-Induced Liver Disease and Female Infertility

**Fig. 3:**
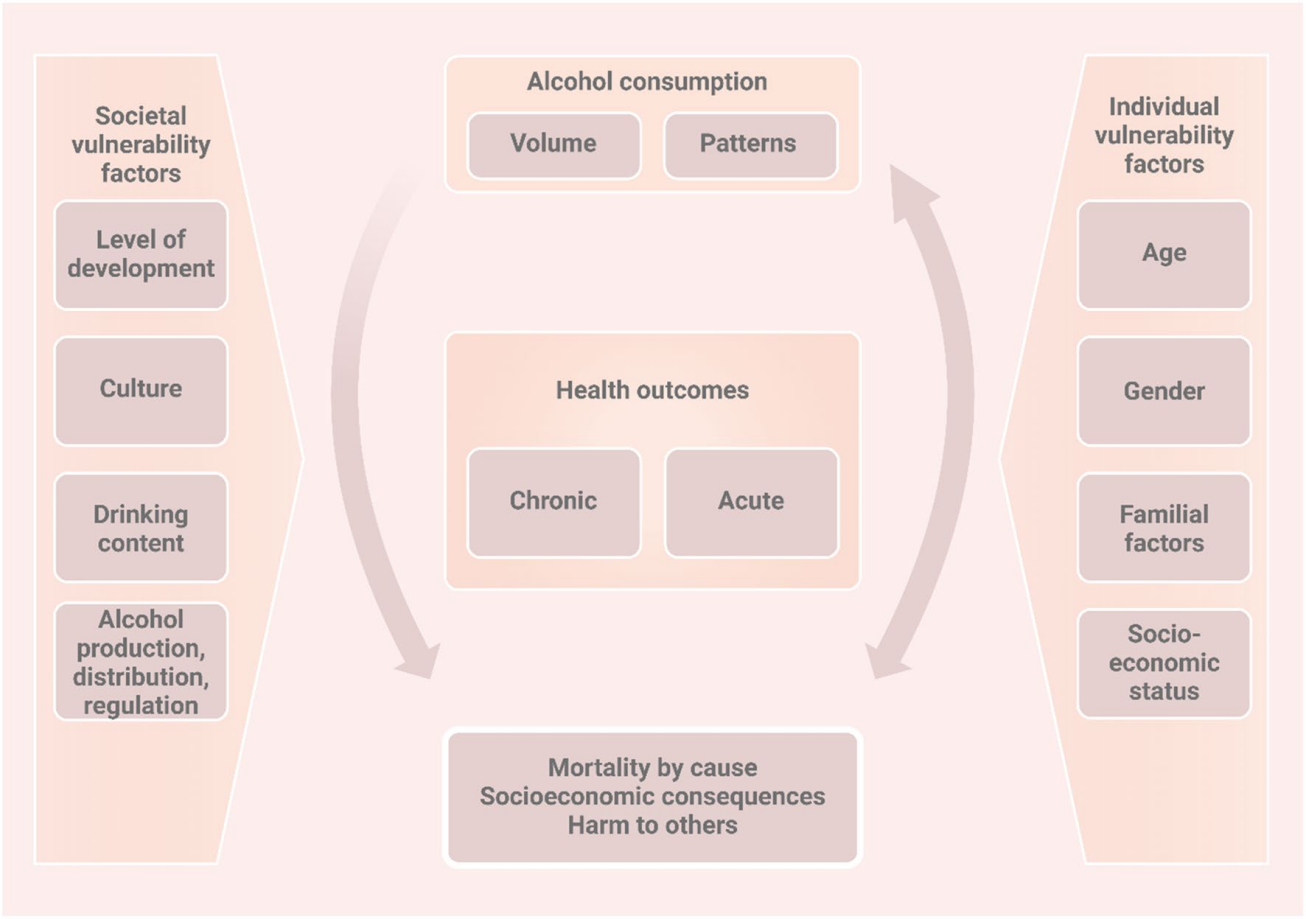
Factors which may control alcohol consumption

An association has been established between chronic alcohol consumption and the onset of liver disease as well as infertility in females [56]. Figure 3 shows some factors that may aid in controlling alcohol consumption. These conditions are caused by complex molecular processes that involve alcohol metabolism, oxidative stress, inflammation, and hormonal imbalances [7]. When considering liver disease, it is observed that females are more susceptible to alcohol-induced liver injury compared to males [57]. This may be owing to variations in the way alcohol is metabolised by women. Since women have lower concentrations of the enzyme ADH in their stomachs and livers, their alcohol metabolism is slower than that of males [58]. Consequently, women have a higher likelihood of developing liver disease even when they consume alcohol at lower amounts compared to men [59]. Chronic alcohol use can cause a number of liver illnesses in women, such as cirrhosis, alcoholic hepatitis, and alcoholic fatty liver disease (AFLD) [7]. The molecular mechanisms that underlie these conditions encompass several critical pathways. Oxidative stress occurs when there is an imbalance between the ability of the body to remove reactive oxygen species (ROS) and the amount of ROS being produced. Chronic alcohol consumption in the liver can lead to the overproduction of reactive oxygen species (ROS), causing harm to lipids, proteins, DNA, and other cellular components [60]. Chronic alcohol consumption can lead to the activation of innate immune components such as Kupffer cells, LPS/TLR4, and complements. These components have a vital role in the development and progression of acute liver illness [61]. Moreover, it has been found that the connection between the gut and liver plays a vital role in the development of alcoholic liver disease (ALD). Extended alcohol consumption triggers the activation of CYP2E1 in liver cells, resulting in compromised antioxidant function, excessive generation of reactive oxygen species (ROS), and increased activity of inducible nitric oxide synthase (iNOS). The reactive oxygen species trigger endoplasmic reticulum stress and generate an inflammatory response through the TLR4/MyD88/NF-κB signalling pathway, which greatly enhances the activation of the NLRP3 inflammasome [61].

**Fig. 4:**
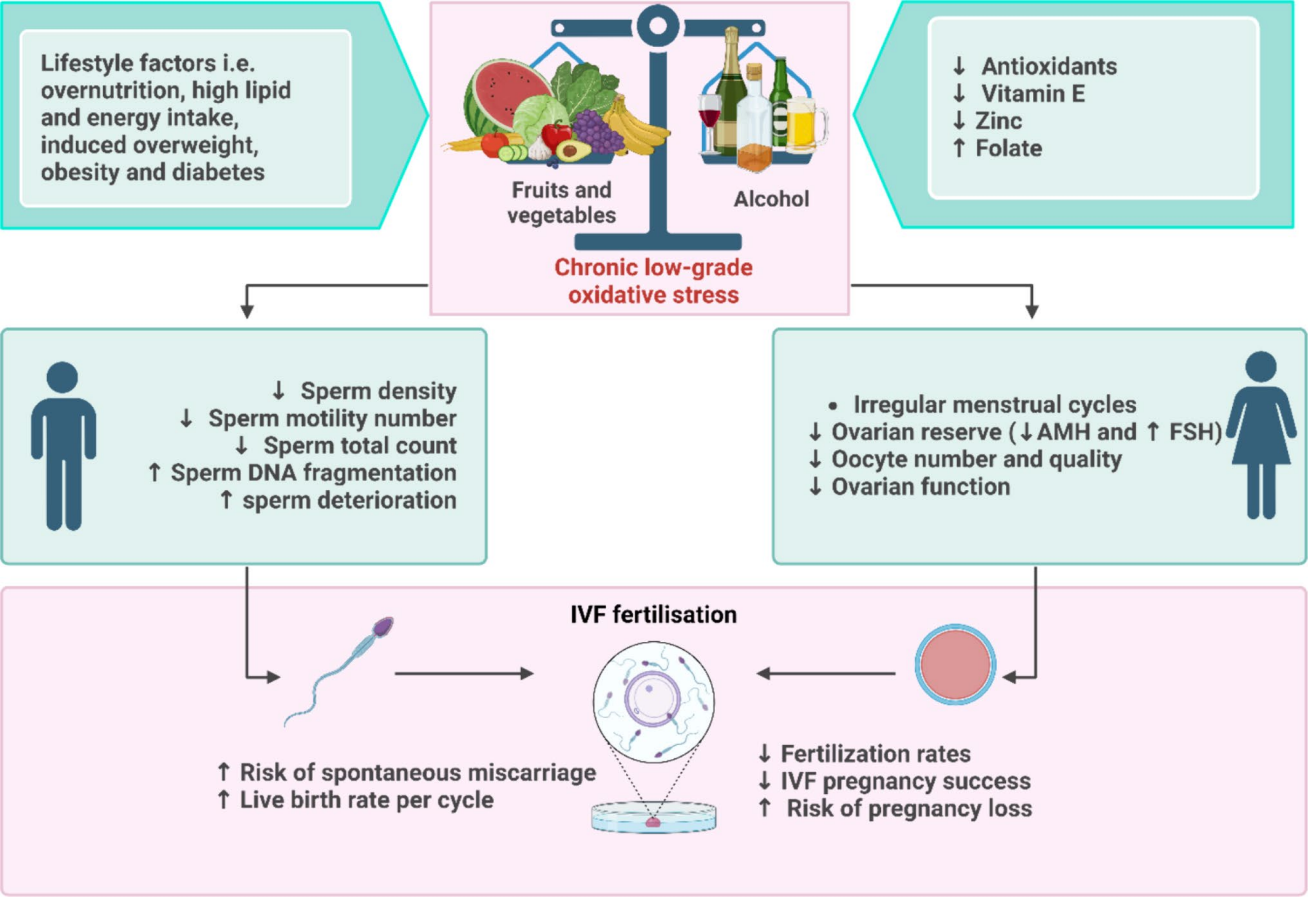
Repercussions of alcohol consumption on the fertility of both men and women

Inflammation has a vital role in the progression of liver disease. Prolonged alcohol consumption has the potential to stimulate inflammatory cells within the liver, which are capable of secreting cytokines and other signalling molecules that have a detrimental effect on tissues and inflammation. Chronic alcohol consumption in relation to female infertility is linked to disturbances in the menstrual cycle, decreased fertility, and an increased risk of miscarriage and foetal alcohol syndrome [62]. The precise molecular mechanisms that contribute to alcohol-induced infertility in women remain poorly elucidated; however, they potentially entail interference with the hypothalamic-pituitary–gonadal axis, a critical regulator of reproductive function [62–67]. Hormonal imbalances caused by chronic alcohol consumption in women may have a negative impact on their fertility [68]. Oestrogen, progesterone, and luteinizing hormone (LH), all of which are essential for ovulation and the menstrual cycle, can be disrupted by alcohol [69]. Additionally, alcohol has the potential to disturb the equilibrium of pregnancy-related hormones, including progesterone and human chorionic gonadotropin (hCG), thereby elevating the likelihood of miscarriage and foetal alcohol syndrome [70]. Figure 4 shows the chronic alcohol consumption has been linked to irregular menstrual cycles, decreased fertility, increased risk of miscarriage, and foetal alcohol syndrome in the context of female infertility [71–73]. Moreover, it is important to remember that smoking and alcohol can both damage DNA and alter the epigenetic makeup of germ cells, which can eventually result in genetic defects and heritable imprinting as well as associated disorders [74, 75] (Table 1).

**Table 1:** Mitigating risks: strategies for promoting moderate alcohol consumption and reducing mortality hazard

Condition	Study performed (animal/human/in vitro)	Type of research (in vitro/in vivo)	Target mechanism	Reference
Breast cancer	Adult female Wistar rats	In vivo model	Nitric oxide synthase	[76]
Breast cancer	MCF-7 and MDA-MB-231 cells Sprague–Dawley female rats BALB/c female mice	In vitro and in vivo	Using *Cinnamomum zeylanicum* L. on MTS, BrdU, cell cycle, annexin V/PI, caspase-3/7, Bcl-2, PARP, and mitochondrial membrane potential analyses	[77]
Breast cancer	MDA-MB-231, MCF7, and T47D Breast epithelial MCF10A and mouse breast cancer cell line 4T1 Female athymic nude mice	In vitro and in vivo	MBZ on CD44 and OCT3/4, and cancer progression-related ESM-1 protein expression in TNBC and RT-R-TNBC cells	[78]
Breast cancer	Tumour and normal breast samples from BC patients BC cell lines MCF-7, T47D, MDA-MB 453, MDA-MB-231, MDA-MB-468, and lung cancer cell line A549 Swiss albino mice	In vitro and in vivo	Nuclear factor erythroid-2 related factor-2 (Nrf2)	[79]
Breast cancer	Human breast carcinoma cell line MDA-MB-231 and mouse breast carcinoma cell line 4T1 Female BALB/c mice	In vitro and in vivo	Caspase-3 and caspase-9	[80]
Breast cancer	Human breast cancer cell lines (MDA-MB-231, MDA-MB-453, MCF-7) Male Sprague–Dawley rats	In vitro and in vivo	AD-1 metabolite M2 (Panaxadiol; PD) on breast cancer cells of nude mice	[81]
Hepatocellular carcinoma	BALB/c nude mice	In vitro model	Endothelial-mesenchymal transition (EMT)	[82]
Oral/oropharyngeal squamous cell carcinoma	Human OSCC cell lines (SCC9 and UM6) Nude mice	In vitro and in vivo model	NFAT Signalling	[83]
Oral cancer	H-357 (oral cancer) and THP-1 (human leukemic monocyte) cell lines Balb/c mice	In vitro, in vivo and ex vivo	Tumour associated macrophages	[84]
Gastric ulcer	RAW264.7 murine macrophage cells Sprague–Dawley rats	In vitro and in vivo	ERK-JNK/c-Jun signal pathways	[85]
Gastric ulcer	Sprague–Dawley rats	In vitro and in vivo	NF-κB, interleukin-1-beta (IL1β) and TNF-α	[86]
Gastric ulcer	Gastric mucosal epithelial cells (GES-1) BALB/c mice	In vitro and in vivo	NF-κB, COX-2, TNF-α, IL-1β and iNOS proteins	[87]
Gastric mucosal injury	RAW 264.7 cells, RGM-1 cells, and BALB/c mice	In vitro and in vivo	Gastroprotection through antioxidant, anti-inflammatory, and anti-apoptotic effects	[88]
Gastric carcinoma	Gastric cancer (AGS) cell lines Wistar albino rats	In vitro and in vivo	PI3K/AKT/mTOR signalling pathway	[89]
Colon tumour	SCID mouse xenograft model	In vitro model	Cell cycle progression	[90]
Colorectal cancer	Humanized PD-L1 knock-In MC38 cancer mouse model	In vivo model	PD-1/PD-L1	[91]
Colorectal cancer	CRC cell lines BALB/c mice	In vitro and in vivo	PD-L1 Expression	[92]
Prostate cancer	TRAMP mice	In vivo model	NRF2 modulation	[58]
Prostate cancer	PC3 prostate cancer cell line BALB/cAnNCrl immunocompromised (athymic nude) mice	In vitro and in vivo	P2X4 Receptor	[93]
Prostate cancer	PC3 and LNCaP cell BALB/c (nu/nu) nude mice	In vitro and in vivo	Tumour necrosis factor-related apoptosis-inducing ligand (TRAIL)	[94]
Prostate cancer	LNCaP and 22Rv1 cells Athymic nude mice (BALB/c nu/nu)	In vitro and in vivo	Androgen receptor signalling	[95]
Brain damage	Sprague Dawley rats	In vivo	PARP inhibitor	[96]
Blood–brain barrier impairment	Rat brain endothelial cell line (RBE4)	In vitro	Blood–brain barrier	[97]
Neuroinflammation	Adult male and female C57BL/6 J mice	In vivo model	IBA-1, CSFR, IL-6, p38 and ERK2/1	[98]
Neuroinflammation and neurodegeneration	Hippocampal-entorhinal cortical (HEC) slices Sprague Dawley rats	In vitro and in vivo	Neuroinflammatory pathways	[99]
Neuroinflammation and neurodegeneration	C57BL/6 mice and mice deficient of MCP-1 (MCP-1 − / −) and CCR2 (CCR2 − / −)	In vivo	MCP-1 and CCR2	[100]
Neurodegeneration	Neural stem cells (NSC) and neuronal-committed progenitors (NCP)	In vitro	NF-кB-, cAMP/PКA-, JAKs/STAT3-, ERK1/2-, p38-pathways	[101]
Neurodegeneration	Sprague Dawley rats	In vivo	Endocannabinoid system	[102]
Neurodegeneration	Wistar rats	In vivo	Transforming Growth Factor-β Group II Metabotropic Glutamate Receptor Activation	[103]
Neurodegeneration	C57BL/6 J Mice	In vivo	CYP2E1	[104]
Neurodegeneration	Sprague–Dawley rats	In vivo	Microglia	[105]
Neurodegeneration	Adult rats	In vivo	Glial activation	[40]
Neurodegeneration	Sprague–Dawley rats	In vivo	AMPK signaling pathway	[106]
Neurodegeneration	Immortalized human microglial cells (HMO6) and human neuroblastoma cells (SH-SY5Y) Human brain tissues	In vitro and in vivo	Protein kinase pathway	[107]
Neurodegeneration	Conventional mice Astrocytes (ACSA-2 + cells), oligodendrocytes (O4 + cells), and microglia (CD11b + cells)	In vitro and in vivo	MAPK ERK1/2 and p38	[108]
Neurodegeneration	Clonogenic PSA-NCAM + cells C57BL/6 mice	In vitro and in vivo	MAPKS ERK1/2 and p38	[41]
Cardiovascular disease	Clonogenic PSA-NCAM + cells C57BL/6 mice	In vitro and in vivo	JAKs and STAT3	[109]
Ischemic stroke	Mice	In vitro and in vivo	Tissue-type plasminogen activator (tPA)	[43]
Ischemic stroke	C57BL/6 J Mice	In vivo	BrdU + /DCX + and BrdU + /NeuN + cells	[110]
Ischemic cerebral injury	PC12 cells Sprague–Dawley rats	In vitro and in vivo	ALDH2	[111]
Cognitive impairment	Wistar rats	In vivo and in silico	Cerebral miRNA155 and NLRP3	[112]
Acute liver injury	Human hepatocytes (LO2 cells) Sprague–Dawley rats	In vitro and in vivo	TLR4/NF-κB signalling pathway	[113]
Liver failure	L-02 cells C57BL/6 mice	In vitro and in vivo	Ferroptosis	[114]
Liver disease	Primary hepatocytes C57BL/6 mice	In vitro and in vivo	Cell elongation 1 (ECE1)	[115]
Liver disease	CD14 + /CD16 + monocytes C57BL/6 J mice	In vivo	Mixed lineage kinase domain‐like pseudokinase (MLKL)	[116]
Liver disease	C57BL/6 mice	In vivo	NF-κB Signaling Pathway	[117]
Liver disease	C57BL/6 mice	In vivo	AIM2 inflammasome activation	[118]
Liver injury	C57BL/6 mice	In vivo	Tnfα, Il6, Mcp1, and Il1β mRNA	[119]
Alcoholic liver disease (ALD)	RAW264.7 cell lines C57BL/6 J mice	In vitro and in vivo	M1 and facilitation of M2 macrophages	[120]
Alcoholic liver injury	Mouse RAW264.7 cells Kupffer cells C57BL/6 J mice	In vitro and in vivo	PTP1B, NF-κB pathway	[121]

## Future Directions: Areas for Further Research and Policy Implications

Policy initiatives and research pertaining to alcohol encompass a vast array of subjects, including public health and social behaviour [122]. Engage in research within the domains of public health and epidemiology to investigate the long-lasting health effects of alcohol intake. This should involve a comprehensive examination of its impact on mental health, chronic illnesses, and overall state of well-being [123]. Policymakers must establish and execute public health initiatives aimed at enhancing knowledge of the detrimental health consequences associated with excessive alcohol consumption [124]. Consider laws that prevent dangerous drinking habits, such as alcohol taxes and advertising limits [125]. Analyse the various determinants that impact the occurrence of underage drinking, the resulting outcomes, and effective strategies for prevention. Establish and uphold rigorous restrictions that govern the sale and distribution of alcohol to those who are under the legal drinking age. Support instructional programmes in schools and communities that aim to reduce underage drinking. Investigate effective interventions and therapies for alcohol consumption disorder, including both pharmaceutical and behavioural approaches. Increase access to high-quality addiction treatment facilities, encourage early intervention, and decrease the stigma associated with getting help for alcohol-related problems [126]. Examine the effects of alcohol on road safety, the efficacy of sobriety checkpoints, and the role of technology in reducing drunk driving. To combat drunk driving, enforce severe regulations, increase public awareness efforts, and invest in technology like ignition interlock devices [127]. Examine the connections between the accessibility of alcohol, the concentration of alcohol-selling establishments, and the negative consequences associated with alcohol consumption. Enforce zoning regulations and licencing prerequisites to restrict the quantity of establishments selling alcohol [128]. Consider pricing and taxation policies to impact alcohol supply and consumption. Investigate how cultural and social norms influence alcohol consumption trends [129]. Create culturally tailored interventions and educational programmes to address social norms and attitudes towards alcohol. Encourage community involvement to promote appropriate drinking habits [126]. Examine the impact of alcohol advertising on patterns of alcohol consumption, specifically among susceptible groups. Enforce regulations on alcohol advertising, specifically targeting the youth demographic [130]. Consider limiting the substance and placement of alcohol marketing to decrease their influence. Investigate harm-reduction methods such as supervised consumption sites and alcohol substitution programmes. Incorporate harm reduction techniques into a comprehensive approach to address alcohol-related problems. Allocate financial resources to initiatives that seek to mitigate the adverse effects of alcohol intake. Investigate the bidirectional link between alcohol use and mental health problems. Integrate mental health care and substance misuse treatment programmes. Enact measures to tackle the simultaneous presence of mental health conditions and substance abuse disorders. Examine the variations in alcohol consumption patterns across different cultures and analyse the resulting effects on policy formulation [131]. To address global alcohol concerns, develop policies that take cultural settings into account and involve international collaboration. Effective policy in these areas should be evidence-based, taking into account the unique requirements of varied populations, as well as individual and social viewpoints on alcohol consumption [132]. A comprehensive approach including numerous stakeholders, including government agencies, healthcare providers, communities, and the alcohol business, is frequently required to make meaningful influence. Alcohol research includes various areas for future research and policy consequences [133, 134].

## Conclusion

The study concludes that alcohol-induced liver disease (ALD) and female infertility are multifaceted health issues with significant societal and individual consequences. Chronic alcohol consumption is strongly associated with increased mortality rates and a range of preventable diseases globally. The molecular mechanisms behind ALD involve a progression from steatosis to fibrosis/cirrhosis, which greatly affects liver function and overall health. Addressing alcohol-related health consequences requires a comprehensive approach involving public health interventions, regulatory measures, and targeted educational programs. Future research efforts should focus on quantifying mortality risks associated with alcohol consumption, establishing causality through longitudinal and epidemiological studies, and understanding gender disparities in alcohol-related health outcomes, particularly regarding female infertility.

## Data Availability

Not applicable.
